# Electrophysiological Examination of Feedback-Based Learning in 8–11-Year-Old Children

**DOI:** 10.3389/fpsyg.2021.640270

**Published:** 2021-02-25

**Authors:** Yael Arbel, Annie B. Fox

**Affiliations:** ^1^Department of Communication Sciences and Disorders, Massachusetts General Hospital, Institute of Health Professions, Boston, MA, United States; ^2^Massachusetts General Hospital, Institute of Health Professions, Boston, MA, United States

**Keywords:** feedback-based learning, feedback-related negativity, event related potentials, development, learning

## Abstract

The study aimed at evaluating the extent to which the feedback related negativity (FRN), an ERP component associated with feedback processing, is related to learning in school-age children. Eighty typically developing children between the ages of 8 and 11 years completed a declarative learning task while their EEG was recorded. The study evaluated the predictive value of the FRN on learning retention as measured by accuracy on a follow-up test a day after the session. The FRN elicited by positive feedback was found to be predictive of learning retention in children. The relationship between the FRN and learning was moderated by age. The P3a was also found to be associated with learning, such that larger P3a to negative feedback was associated with better learning retention in children.

## Introduction

Learning from feedback is an important ability, particularly during the early school years when children are required to adapt to a structured and demanding learning environment where performance is frequently evaluated and corrected. The ability to utilize external feedback efficiently can be viewed as a task of the executive control system that goes through a maturation process throughout childhood (Welsh et al., 1991; Casey et al., 1997; Adleman et al., 2002; Perner and Lang, 2002; Rubia et al., 2007; Vijayakumar et al., 2014). Indeed, the notion that learning from feedback goes through developmental changes from childhood to adulthood is supported by behavioral and neurophysiological evidence. For example, children have been reported to rely more heavily and respond more strongly to external feedback when compared with adults (Eppinger et al., 2009; Hämmerer et al., 2011). There are also reports that children differentiate less between positive and negative feedback (Hämmerer et al., 2011; Mai et al., 2011; Zottoli and Grose-Fifer, 2012), and are more susceptible to interference from uninformative (Crone et al., 2004) or deceptive feedback (Eppinger et al., 2009). To date, feedback processing in children has been studied using paradigms focused primarily on probabilistic learning (e.g., Eppinger et al., 2009) and reward processing (e.g., van Leijenhorst et al., 2006; Crowley et al., 2013). Because children are frequently engaged in declarative learning (i.e., intentional acquisition of knowledge by building concept-associations in memory) as part of their schooling, it is important to shed light on their ability to use feedback to facilitate such learning. The present study was designed to evaluate the use of external feedback by school-age children to support declarative learning.

### Electrophysiological Measures of Feedback Processing

The study of feedback processing in children has been advanced by the discovery of an electrophysiological marker of feedback processing (Miltner et al., 1997). The feedback related negativity (FRN), is an event related potential (ERP) elicited by feedback stimuli delivered in various learning (e.g., Pietschmann et al., 2008; Krigolson et al., 2009; Sailer et al., 2010; van der Helden et al., 2010; Ernst and Steinhauser, 2012; Arbel et al., 2013, 2014; Luft, 2014) and gambling paradigms (e.g., Gehring and Willoughby, 2002; Hajcak et al., 2007; Goyer et al., 2008). The FRN is a negative going ERP with a peak amplitude at about 250–300 ms following the presentation of a feedback stimulus. Its amplitude is maximal over fronto-central recording sites, and it is typically larger for negative feedback than positive feedback. The anterior cingulate cortex (ACC) has been suggested to be the generator of the FRN (Botvinick et al., 2001; Nieuwenhuis et al., 2004; Yeung et al., 2004) through a phasic change in dopaminergic input projected to the basal ganglia and ACC (Holroyd and Coles, 2002). It has been suggested that the activation associated with the FRN is driven by increased inhibition of ACC following positive feedback (Holroyd et al., 2008; see Proudfit, 2015 for review). Although the study of the FRN has focused primarily on the activation associated with negative feedback, evidence suggests that in some paradigms the processing of positive feedback drives the observed sensitivity of the FRN to experimental conditions (Holroyd et al., 2008; Eppinger et al., 2009; Baker and Holroyd, 2011; Foti et al., 2011; Kreussel et al., 2012; Luque et al., 2012; Arbel et al., 2013). In line with this evidence, an alternative view of the FRN as a positivity triggered by reward rather than a negativity elicited by negative feedback has been proposed. The Reward Positivity (RewP) is suggested to be triggered by the processing of reward, suppressed by losses or negative feedback and generated in the striatum (e.g., Holroyd et al., 2008; Foti et al., 2011; Proudfit, 2015; Kujawa et al., 2018). Larger RewP has been reported to be associated with higher self-report scores on the Reward Responsiveness Scale (Bress et al., 2012), and with a greater response bias to make richly rewarded decisions (Bress and Hajcak, 2013). This alternative interpretation of the FRN affects the discussion of its functional significance. The open discussion on whether the FRN is a negativity sensitive to negative feedback or a positivity sensitive to rewards calls for measuring activations associated with positive and negative feedback separately rather than treating one as a “baseline” that can be subtracted from the other.

A Fronto-central positivity, which proceeds the FRN, is another ERP component associated with feedback processing. This positivity has a peak latency of about 350–400 ms at fronto-central recording sites and is typically larger for negative feedback. Its spatial and temporal distribution suggests that this fronto-central positivity is a P3a, linked to the initial processing of a novel stimulus (Spencer et al., 1999), and assumed to index attentional orienting (Anderson, 2002; Butterfield and Mangels, 2003, Romine and Reynolds, 2005; Conklin et al., 2007) with increased amplitude related to greater focal attention (Polich, 2007). In the context of feedback processing, it has been found sensitive to valence, with larger amplitude to negative feedback (e.g., Butterfield and Mangels, 2003; West et al., 2014, 2018; Wischnewski et al., 2018) and learning outcomes (Arbel and Wu, 2016; Wischnewski et al., 2018; Valt et al., 2019). In a feedback-based two-choice declarative learning paradigm (Arbel and Wu, 2016) larger P3a to negative feedback in the first training block predicted better learning outcomes on a test at the conclusion of the task. In a decision-making task with advice cues of different predictive levels, the P3a component was found to be influenced by the subjective predictive value of an advice cue, with the most informative expert cues showing the largest P3a amplitude (Wischnewski et al., 2018). In a probabilistic task aimed at examining performance evaluation in relation to performance of another, the P3a was found sensitive to relative performance such that a larger P3a was observed when performance of self was worse than performance of another (Valt et al., 2019). The suggested interpretation was that this relatively poor performance called for the recruitment of attentional resources (Valt et al., 2019).

### ERPs in the Study of Developmental Changes in Feedback Processing

There is a growing interest in the developmental changes in feedback processing and in the FRN as a possible tool to evaluate these changes. Using the FRN for such evaluation is permitted by evidence that the FRN is reliably identified and measured in young children (e.g., van Meel et al., 2012), and even in toddlers (Meyer et al., 2014; Roos et al., 2015). When comparing the FRN in children with that of adolescents and adults, a common reported pattern is a larger FRN in children, regardless of feedback valence (Hämmerer et al., 2011; Zottoli and Grose-Fifer, 2012; Crowley et al., 2013; Ferdinand et al., 2016; Arbel et al., 2017), with some studies reporting no age related differences in FRN amplitude (Santesso et al., 2011; Yi et al., 2012; Lukie et al., 2014). Age-related decrease in FRN amplitude is interpreted to reflect an excessive reliance on external feedback at a young age that is decreasing with time as a function of the gradual maturation of the executive control system. While a growing body of literature has established a connection between the FRN and learning in adults (e.g., Pietschmann et al., 2008; Krigolson et al., 2009; Van den Bos et al., 2009, see review by Eppinger et al., 2009; Sailer et al., 2010; van der Helden et al., 2010; Arbel et al., 2013, 2014; Luft, 2014), very few reports are available on the relationship between the FRN and learning in children. In a report by Eppinger et al. (2009), the FRN elicited by feedback of varying validity was examined in a probabilistic task completed by 10–12-year-old children and adults. The results suggested that learning and FRN patterns were affected by the presence of invalid feedback in children but not in adults. In children, the presence of occasional invalid feedback was associated with a diminished amplitude difference between FRN to positive and negative feedback. In a study by Groen et al. (2007) who employed a stimulus-response mapping task with probabilistic feedback, no FRN was observed in 10–12-year-old children. The authors suggested that the absence of the FRN may have stemmed from the limited motivational salience of the feedback stimuli used in their paradigm. Shephard et al. (2014) compared performance and FRN patterns in 9–11-year-old children and adults who performed an S-R mapping learning task with consistent (non-probabilistic) performance feedback. Their examination of the FRN elicited by positive feedback in consecutive blocks of the learning task suggested a reduction in the negativity of the FRN peak amplitude to positive feedback as learning progressed. Meyer et al. (2014) studied the FRN in toddlers who played a feedback-guided paired-associate game on a touchscreen. The amplitude difference between the FRN elicited by positive feedback and FRN elicited by negative feedback was found predictive of adaptive behavior, with greater FRN amplitude differences associated with more adaptive performance. While these studies provide evidence that the FRN reflects developmental changes in the processing of positive and negative feedback in the context of reward processing, probabilistic learning and S-R mapping, the extent to which this signal reflects the use of feedback for declarative learning in children is yet to be examined.

### The Present Study

The aim of the study was to evaluate the relationship between feedback processing as measured by the feedback related ERPs and declarative learning in children. In a previous study (Arbel and Wu, 2016), the FRN and P3a were found sensitive to learning outcomes when healthy young adults completed a two-choice declarative learning task. More specifically, small FRN to negative feedback, large FRN to positive feedback, and large P3a to negative feedback predicted successful learning. The hypotheses of the present study were based on these findings, and on current understanding of feedback processing in children. We expected that, in children, the FRN to positive feedback will be found related to learning outcomes, and that such relationship will be weaker for negative feedback because of the assumed immature ability to extract task relevant information from negative feedback in children. Based on the interpretation of the P3a as reflecting level of attention to the feedback, and in light of previous findings in adults we anticipated that larger P3a to negative feedback will be associated with better learning outcomes. The selected age range of 8–11 years represents an important period in the development of executive functions, as a significant shift to adult level performance in various executive functions, particularly in feedback processing, is reported to begin within the selected age range (e.g., Van Duijvenvoorde et al., 2008). Moreover, previous reports of differences in brain activation related to feedback processing between children who are 8–9 years old and those who are 11–13 years old (e.g., Crone et al., 2004; van Duijvenvoorde and Crone, 2013) shaped our expectations to find age-related differences in feedback processing even in the narrow age range of 8–11 years. More specifically, we hypothesized that the youngest children in our sample would demonstrate large FRN to negative feedback, but that this heightened sensitivity to feedback valence will not be associated with learning outcomes, indicating an immature processing of negative feedback.

## Methods

### Participants

Data of eighty children between the ages of 8;0 and 11;0 years (mean age 9;5 years, SD, 1.07; 41 females, 39 males) who participated in a larger scale longitudinal study, whose aim is to evaluate developmental changes in feedback-based learning, were used for the reported project. Participants had normal or corrected to normal vision, had no history of developmental or acquired neurological disorders, and English was reported to be their primary language. The study was approved by the Partners HealthCare IRB. Data collection began after assent was obtained from the participant and the parents signed a consent form. Participants were monetarily compensated for their time.

### Procedure

#### Task

Each participant sat in a comfortable chair about 60 cm from a computer monitor and completed a declarative word learning task. Participants were tasked with learning the names of 20 novel objects. In each trial, participants viewed pictures of two novel objects on a computer screen coupled with a name presented auditorily through speakers. While the location of the two objects on the screen (right and left) changed randomly throughout the task, the same pairs of objects were coupled with each name. Participants were instructed to choose the picture of the novel object that corresponded to the name. Responses were made by pressing one of two buttons on a response box. Participants were allotted 5,000 ms to respond, and their response was followed by a blank screen for 500 ms which was proceeded by visual feedback (“√*√√*” for correct responses and “xxx” for incorrect responses) presented for 1,000 ms to indicate the correctness of the response. Each set of two novel objects and a name were presented once in each block of trials, for a minimum of five blocks of trials, and a maximum of ten blocks of trials. Within this range, number of blocks (number of repeated presentations of the stimuli) were determined based on the achievement of a learning criterion of reaching a cumulative accuracy level of more than 90%. Accuracy evaluation for the purpose of determining the cumulative accuracy level began on the fourth block. During the first presentation of the stimuli (first block) participants' responses were followed by equally probable negative and positive feedback (0.5 positive, 0.5 negative) to ensure that all participants begin the learning experience with the same amount of correct and incorrect responses. Associations between objects and names were created by the E-Prime program based on participants' responses during the first block and were kept throughout the remainder of the task. Consequently, associations between objects and names varied across participants. Participants received feedback, which was consistent with their performance throughout the task. At the conclusion of the task, participants were presented with a test block where each combination of objects and names was presented once for a total of 20 trials, without performance feedback. About 24 h following task completion, participants completed a follow up test during which they were presented with the objects and names in the same manner they were exposed to during the test block of the experiment and were tasked with selecting the object that corresponded with the name they heard through computer speakers. E-Prime 2® by Psychology Software Tools (PST) was used for task preparation and presentation and the Chronos® response device by PST was used to record responses.

##### Stimuli

Black and white illustrations of the novel objects were borrowed from Kroll and Potter (1984). Non-words were produced from the ARC Non-word Database (Rastle et al., 2002). The non-words were monosyllabic, in four letter C_1_V_1_C_2_C_3_ format (e.g., “ZIMF”) and phonologically legal in English. The number of phonological neighbors were set to be below twenty to create a list of words that are not similar in their pronunciation to other words in English.

#### EEG Data Acquisition and Signal Processing

The Electrical Geodesics Inc. (EGI; Eugene, OR) System 400 with 32-channel HydroCel Geodesic Sensor Nets from EGI was used to acquire and analyze electroencephalogram (EEG) data. The EEG was continuously recorded at a 1,000 Hz sampling rate with a vertex reference, and electrode impedances were kept below 50 kΩ, which is the manufacturer's recommended impedance threshold for this system. EEG data were filtered using an offline bandpass of 0.1–30 Hz filter, and then segmented into epochs, each starting 200 ms before the feedback presentation and ending 800 ms after the feedback. Epochs containing amplitudes > ±75 μv were rejected, leading to an exclusion of up to 0.1 of the segments across all participants. Independent Component Analysis (ICA) was performed on the data to detect and reject factors that account for artifacts of eye-blinks and eye movement (Delorme and Makeig, 2004). On average, participants were presented with 184 (*SD* = 34) trials, of which an average of 142 (*SD* = 35) trials received positive feedback, and an average of 43 (*SD* = 23) trials received negative feedback. All participants had a minimum of 20 segments per condition at FCz and after artifact rejection and ICA. Averages of the artifact-free epochs were calculated for each feedback type (positive and negative feedback), after baseline correcting each average over the 200 ms pre-feedback baseline. The averaged EEG epochs were re-referenced to the average of all electrodes.

#### ERP Data Analysis

Visual inspection of the fronto-central electrodes determined that the FRN and the positivity that followed it (P3a) were maximal at FCz. Electrode FCz was therefore chosen for the analysis of the FRN and P3a ERP components. Data from electrode *FCz* across feedback types and participants were entered into a temporal PCA (TPCA) to reduce the temporal dimensionality of the dataset (e.g., Arbel and Wu, 2016; Arbel et al., 2017), using *Promax* rotation (Dien, 2010), after correcting for latency variance. Latency correction was performed by aligning the peak latencies of the FRN and P3a of individual averages with the peak latency of the grand average waveform (Brumback et al., 2012; Kim and Arbel, 2019). The analysis used the covariance between time points and resulted with a set of seven temporal factors accounting for 90% of the total variance. Temporal factor 4 with a maximal peak around 250–300 ms was identified as capturing the FRN activation. The P3a was depicted by temporal factor 3 (peak latency of 380–400 ms). The factor scores of temporal-factor 4 (FRN) and temporal factor 3 (P3a) were the amplitude measures of each of the components of interest and were entered into the statistical analyses.

### Statistical Analysis

We examined whether the FRN and P3a to positive and negative feedback predict learning retention in children. Retention was measured as the number of correct responses on the learning task 24 h after completing the declarative learning task. Three participants were removed from the analysis of the learning retention outcome due to missing data, bringing the final samples to *n* = 77 for retention.

Because the learning retention outcome was the number correct out of 20 trials (i.e., either correct or incorrect for each trial), we initially used a generalized linear model with a binomial distribution and a logit link function to analyze the relationships between FRN and P3a with learning retention. The analysis was conducted separately for the FRN and P3a. Models were first tested with just the FRN or P3a for positive and negative feedback included as predictors. Then age and the interactions with FRN or P3a were added to the model to determine if the relationship between the FRN and P3a with learning varied as a function of age. All analyses were conducted using R version 3.5.1 with packages “Stargazer” (Hlavac, 2018), “MASS,” and “robustbase” (Maechler et al., 2019).

An examination of the regression diagnostics of preliminary models indicated potential heteroscedacticity of the residuals and several outliers/influential cases (e.g., large Cook's distance or leverage values). Rather than remove cases from the analysis, we tested models using robust methods that de-weighted outliers (*lmrob* and *glmrob* from the “robustbase” package). Both *lmrob* and *glmrob* use iteratively reweighted least squares (IRLS) and deweight cases based on the size of the residuals. Below we present the results of the robust analyses. Predictor variables (age, FRNs, and P3a) were standardized (z-scores) prior to analysis for all models we tested. The model coefficients presented in the tables below are therefore equivalent to partially standardized coefficients which can also be interpreted as effect sizes.

## Results

### Behavioral Data

Accuracy and reaction time data across training blocks are presented in Table 1. Participants' average number of incorrect responses before reaching the learning criterion of a cumulative accuracy level of more than 90% during training was 43 errors (*SD* = 23). Repeated measures ANOVA on accuracy across training blocks yielded a Block effect, *F*_(1, 9)_ = 73.24, *p* < 0.0001, indicating that participants' accuracy increased as the task progressed. Age was found negatively correlated with number of errors, *r* = −0.37, *p* < 0.001, suggesting that as age increases, learning becomes faster. Repeated measures ANOVA of reaction time associated with correct and incorrect responses across blocks was also conducted. A Block effect was found, *F*_(1, 9)_ = 4.54, *p* < 0.0001, indicating an overall reduction in response time throughout the training blocks. No Accuracy effect was found, *F*_(1, 105)_ = 1.38, *p* = 0.24, indicating that reaction time did not differed between correct and incorrect responses. However, an interaction between Block and Accuracy was found, *F*_(1, 9)_ = 2.52, *p* = 0.021. We followed this analysis with separate repeated measures ANOVAs for correct and incorrect trials. The analyses indicated that whereas a Block effect was present for correct trials, *F*_(1, 9)_ = 21.49, *p* < 0.0001, it was absent for error trials, *F*_(1, 9)_ = 0.82 *p* = 0.58. These results indicate that correct responses became faster throughout the training blocks, whereas incorrect responses did not change significantly over time. Correlational analysis between age and RT differences between early and late correct and incorrect trials resulted in no significant correlations, *r* = 0.11, *p* = 0.31 (correct trials), *r* = 0.12, *p* = 0.28 (errors trials).

**Table 1:** Accuracy (proportion correct) and reaction time in milliseconds of correct and incorrect responses across the training blocks excluding block 1 in which accuracy was set to 0.5.

	**Block 2**	**Block 3**	**Block 4**	**Block 5**	**Block 6**	**Block 7**	**Block 8**	**Block 9**	**Block 10**
Accuracy (SD)	0.59 (0.13)	0.65 (0.14)	0.71 (0.16)	0.74 (0.17)	0.79 (0.17)	0.80 (0.16)	0.81 (0.15)	0.83 (0.15)	0.83 (0.15)
RT correct (SD)	1551.66 (388.69)	1432.63 (336.98)	1376.11 (307.07)	1332.63 (326.61)	1285.397 (258.81)	1237.968 (250.02)	1218.263 (267.49)	1259.096 (324.05)	1267.173 (323.53)
RT error (SD)	1592.20 (495.68)	1544.72 (435.53)	1498.74 (406.96)	1536.61 (462.91)	1431.53 (481.82)	1397.03 (532.42)	1373.66 (451.97)	1351.18 (530.66)	1421.11 (495.89)

Participants' average accuracy on the immediate test was 0.89 (*SD* = 0.14), and 0.83 (*SD* = 0.14) on the follow-up test. Performance on the follow-up test served as our measure of learning retention. The accuracy rate of two participants was below chance on the two tests. An examination of their performance during the training phase suggested that they have learned the names of a subset of non-objects (five items at an accuracy rate >66% for one of the learners, and seven items for the other learner), indicating that some learning took place, and that performance was not random. For this reason, the two participants were not excluded from the analysis. Correlation analyses demonstrated that age was positively correlated with learning retention, *r* = 0.27, *p* = 0.016, suggesting that as age increases, learning retention also increases.

### ERP Data

**Figure 1:**
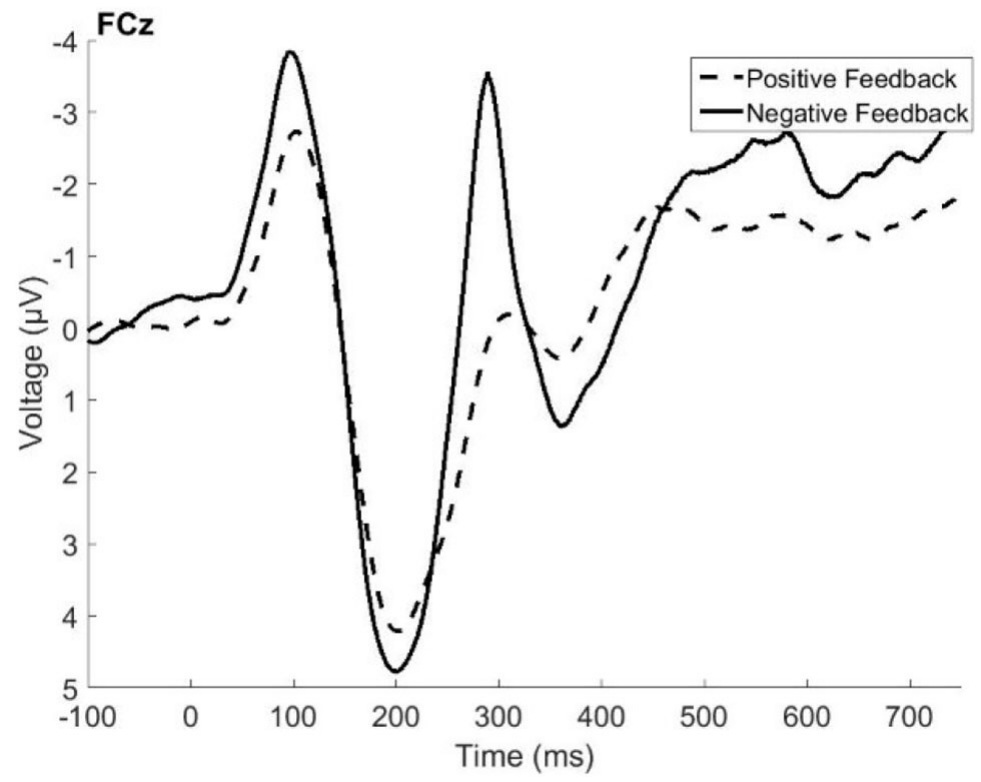
Grand average event related potentials elicited by positive feedback (dashed line) and negative feedback (solid line) recorded in the fronto-central electrode site (FCz).

Figure 1 presents the grand average ERP data from electrode *FCz*, where the FRN and P3a were found to be the largest. Visual inspection of the figure indicated that the FRN peaked at about 287 ms following the feedback, and that its amplitude was^1^ larger (more negative) for negative feedback when compared with positive feedback. The difference in amplitude between positive and negative feedback approached significance, *t*_(79)_ = −1.96, *p* = 0.05. The P3a peaked at around 350 ms following the feedback, and its amplitude is larger following negative feedback, *t*_(79)_ = 3.85, *p* = 0.0002. The results described below are the product of a temporal PCA on electrode FCz, with the factor scores of temporal factor 4 (maximal peak around 250–300 ms) and temporal factor 3 (time window of peak latency of 380–400 ms) serving as the amplitude measures of the FRN and P3a, respectively.

#### FRN

**Figure 2:**
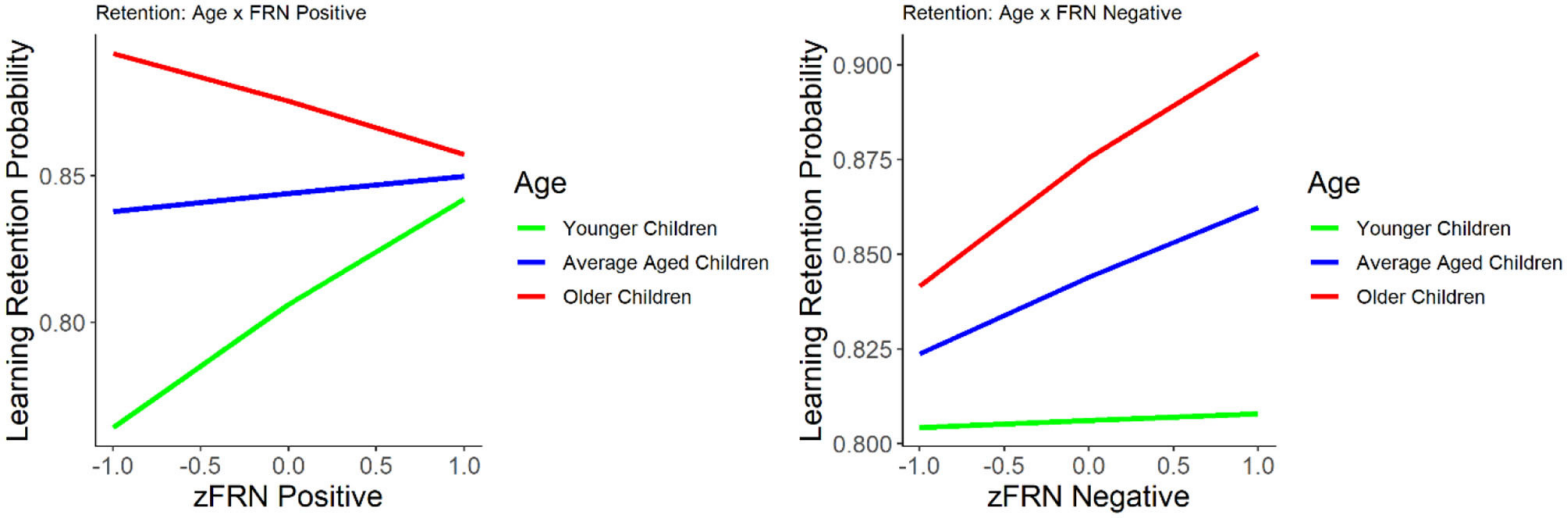
The relationship between learning retention and the FRN elicited by positive (left) and negative (right) feedback. Learning retention is represented by the proportion of correct test items. The FRN amplitude is presented in z score.

We first evaluated the relationship between the FRN and the number of errors committed before reaching a learning criterion. The results suggest that the amplitude of the FRN to positive feedback was negatively correlated with number of errors, *r* = −0.27, *p* = 0.016, indicating that smaller (less negative) FRN to positive feedback was associated with the commission of fewer errors. No significant correlation was found between FRN to negative feedback and number of errors, *r* = −0.19, *p* = 0.08. We explored the nature of the interaction using the *effects* package in R (Fox, 2003; Fox and Weisberg, 2018, 2019). Using the model and relying on the mean age and ±1 SD from the mean, we broke down the age category into three groups: younger (*M* = 8.4 years), average (*M* = 9.5 years), and older children (*M* = 10.5 years), and then plotted the slope of the FRN for positive feedback for each of the three groups (see Figure 2). The interaction indicates that the relationship between a small FRN to positive feedback and smaller number of errors before reaching the learning criterion was present in younger children in our sample but not in older children.

#### FRN and Learning Retention

The results of the GLM model examining the FRN for positive and negative feedback as predictors of learning retention are presented in Table 2. The FRNs for positive and negative feedback were not significant predictors of learning retention, with or without age in the model, *p*'s > 0.09. Age was a significant and positive predictor of learning retention, *b* = 0.25, *SE* = 0.08, *p* = 0.002, indicating that increase in age was associated with better learning retention. The interaction between age and the FRN for negative feedback was not statistically significant, *p* = 0.16. However, the interaction between age and the FRN for positive feedback was statistically significant, *b* = −0.305, *SE* = 0.11, *p* = 0.004. We explored the nature of the interaction using the *effects* package in R (Fox, 2003; Fox and Weisberg, 2018, 2019). While age was treated as a continuous variable in all of our analyses, for the purposes of visualizing the interaction (Figure 2), we graphed the relationship between FRN and learning retention at three different ages: mean (*M* = 8.4 years), one SD above the mean (10.5 years), and one SD below the mean (8.4 years). The interaction suggests that while smaller FRN (less negative) to positive feedback was associated with better learning retention in young children in our sample, whereas in older children larger FRN (more negative) to positive feedback was associated with better retention.

**Table 2:** FRN and age as predictors of learning.

	**Learning retention (Robust GLM)**	
	**Without age**	**With age**
FRN negative feedback (standard error)	0.196 (0.115)	0.112 (0.114)
FRN positive feedback	0.038 (0.112)	0.098 (0.116)
Age		0.247^**^ (0.080)
Age × FRN negative feedback		0.152 (0.109)
Age × FRN positive feedback		−0.305^**^ (0.106)

** *p < 0.01; Numbers presented in the table represent partially standardized regression coefficients, as all predictors were standardized prior to analysis. Standard errors are presented in parentheses; GLM, Generalized Linear Model with binomial distribution*.

#### P3a

We first evaluated the relationship between the P3a and the number of errors committed before reaching a learning criterion. The results suggest that the amplitude of the P3a to positive feedback was not correlated with number of errors, *r* = 0.03, *p* = 0.77. A significant correlation was found between P3a to negative feedback and number of errors, *r* = −0.36, *p* = 0.001, indicating that larger P3a to negative feedback was associated with a smaller number of errors.

#### P3a and Learning Retention

**Figure 3:**
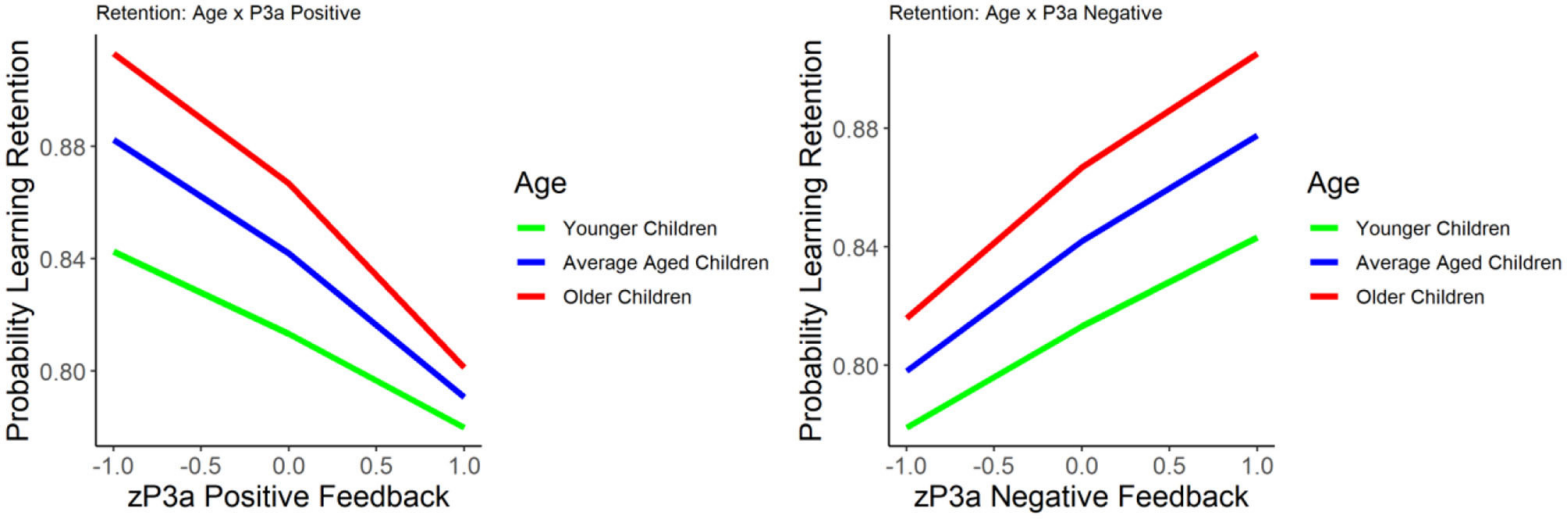
The relationship between learning retention and the P3a elicited by positive (left) and negative (right) feedback. Learning retention is represented by the proportion of correct test items. The P3a amplitude is presented in z score.

The results of the GLM models examining the P3a for positive and negative feedback as predictors of learning retention are presented in Table 3. The P3a for negative feedback was positively associated with learning retention, *b* = 0.422, *SE* = 0.09, *p* < 0.001, such that larger P3a to negative feedback was associated with better learning retention. The P3a for positive feedback was significantly negatively related to learning retention, *b* = −0.436, *SE* = 0.09, *p* < 0.001, indicating that smaller P3a to positive feedback was associated with better learning retention. When age was entered into the model, age was significantly related to learning retention, *b* = 0.216, *SE* = 0.09, *p* = 0.02, as were the P3a for negative feedback (*b* = 0.327, *SE* = 0.105, *p* < 0.01) and positive feedback (*b* = −0.4, *SE* = 0.09, *p* = 0.002). The interactions between age and the P3a for positive and negative feedback were both non-significant, *p*'s = 0.40 and 0.17, respectively (see Figure 3).

**Table 3:** P3a and age as predictors of learning.

	**Learning retention (Robust GLM)**	
	**Without age**	**With age**
P3a negative feedback (standard error)	0.422^***^ (0.091)	0.327^**^ (0.105)
P3a positive feedback	−0.436^***^ (0.088)	−0.400^***^ (0.093)
Age		0.216^*^ (0.093)
Age × P3a negative feedback		0.085 (0.102)
Age × P3a positive feedback		−0.129 (0.094)

* *p < 0.05*,

** *p < 0.01*,

*** *p < 0.001*;

*Numbers presented in the table represent partially standardized regression coefficients, as all predictors were standardized prior to analysis. Standard errors are presented in parentheses; GLM, Generalized Linear Model with binomial distribution*.

### Summary of Results

The FRN elicited by positive feedback was found to be predictive of learning retention in children. Interaction effects indicated that the relationship between FRN and learning were moderated by age, such that sustained learning was predicted by small FRN (less negative) to positive feedback among young children in the sample, while in older children, larger FRN (more negative) to positive feedback was associated with better learning retention. P3a elicited by positive and negative feedback was also found to be associated with learning retention, such that smaller P3a to positive feedback and larger P3a to negative feedback were associated with sustained learning.

## Discussion

The present study evaluated the relationship between the feedback-related ERPs and declarative learning in children aged 8–11 years. Previous findings, using a similar learning paradigm with healthy young adults, suggested that the FRN and P3a elicited to feedback are associated with learning in adults, such that small (less negative) FRN to negative feedback, large FRN (more negative) to positive feedback and large P3a to negative feedback were associated with strong learning (Arbel and Wu, 2016). In the present study, the FRN and P3a were found associated with learning retention in children. While no relationship was found between FRN to negative feedback and learning in our sample, FRN elicited by positive feedback was associated with learning retention. These findings of no sensitivity of FRN to negative feedback to learning in children may reflect an immature ability to take advantage of negative feedback for learning, supporting previous evidence of developmental differences in the ability to process negative feedback. Such differences have been reported by Van Duijvenvoorde et al. (2008) who collected fMRI data while participants completed a feedback-based rule selection and application task. Compared with adults, the performance of 8- to 9-year-old was found to be negatively affected by negative feedback. Additionally, Van Duijvenvoorde et al. (2008) reported different patterns of brain activation for young adults and children, with greater activation in the dorsolateral prefrontal cortex after negative feedback than after positive feedback in adults but not in children. Another developmental fMRI study was conducted by Peters et al. (2014) who examined the age at which the processing of negative feedback reaches the adult level by tasking 8–17-year-old children with using performance feedback to correctly sort animals into one of three squares. The results indicated an increase in the activation of the Anterior Cingulate Cortex following negative feedback until the age of 14 years, and stabilization thereafter. It is important to note that, similar to our study, the performance feedback used in Peters et al. (2014) and Van Duijvenvoorde et al.'s (2008) learning paradigms was informative and deterministic, such that learning was guided by a trial-by-trial feedback that was consistent with participants' responses. Additional evidence for the inefficient processing of negative feedback by children comes from studies in which negative feedback varied in its validity (Crone et al., 2004; Eppinger et al., 2009). In these studies, adults' data indicated that they recognized invalid negative feedback as irrelevant for learning, evidenced by their accuracy and electrophysiological data. Children, on the other hand, processed invalid feedback similarly to valid feedback (Crone et al., 2004; Eppinger et al., 2009), indicating inefficiency in using negative feedback to facilitate learning.

Our data indicate that the amplitudes of the FRN elicited in response to positive feedback was associated with learning in children, such that FRN to positive feedback predicted learning retention. The association between the processing of positive feedback and learning is in line with evidence of greater modulation of FRN to positive feedback (Cohen et al., 2007; San Martin et al., 2010; Baker and Holroyd, 2011; Foti et al., 2011; Kreussel et al., 2012; Arbel et al., 2013), and with previous reports of a relationship between the FRN (Arbel et al., 2013, 2014; Shephard et al., 2014, 2016) and P3a (Arbel and Wu, 2016) elicited to positive feedback and learning. For example, in a feedback-based four-choice word learning task presented to healthy young adults, ERPs associated with positive feedback were found sensitive to subsequent learning, such that words that were subsequently recalled elicited larger FRN (more negative) to positive feedback during the learning process (Arbel et al., 2013), suggesting that heightened response to positive feedback facilitates learning. Similarly, Arbel and Wu (2016) reported that large FRN to positive feedback was found associated with better learning (Arbel and Wu, 2016). Studies that evaluated the change in FRN amplitude during the learning process reported a reduction in FRN amplitude to positive feedback as leaning progresses (Arbel et al., 2014; Shephard et al., 2014). For example, Shephard et al. (2014) reported a reduction in the negativity of the FRN to positive feedback throughout the learning blocks in children performing an S-R mapping task with deterministic feedback. In a two-choice word learning declarative task performed by adults, the amplitude of the FRN to positive feedback also showed a reduction in negativity over the learning process, while the amplitude of the FRN to negative feedback remained constant (Arbel et al., 2014), suggesting that while negative feedback continues to be informative as learning progresses, positive feedback loses its importance as a facilitator of learning when learning is established. These results can also be interpreted within the context of the expectancy account of the FRN (e.g., Oliveira et al., 2007; Bellebaum and Daum, 2008; Ferdinand and Opitz, 2014) to suggest that positive feedback becomes better anticipated as learning progress. The data of the present study point to age related interactions between FRN to positive feedback and learning. Although the age range in our sample is relatively small, significant age-related differences in the relationship between the processing of positive feedback and learning were found. For the younger children in our sample (around the age of 8 years), smaller FRN to positive feedback was associated with better learning. Older children in our sample (around the age of 11 years) showed an opposite pattern, with larger FRN to positive feedback being associated with better learning. The association between the FRN to positive feedback and learning among the older children in our sample is similar to that previously reported in healthy young adults where large FRN to positive feedback was found associated with better learning (Arbel et al., 2013; Arbel and Wu, 2016). The sensitivity of positive feedback to learning in our sample calls for the interpretation of the results based on the view of the FRN as a Reward Positivity (RewP). Within this context, our data indicate that in younger children, larger RewP to positive feedback predicted learning outcomes. These results may suggest that younger children are more responsive to positive feedback and that this sensitivity is important for their learning.

In the present study, the P3a elicited by positive and negative feedback was found to be associated with learning, such that larger P3a to negative feedback was associated with better learning. In light of Butterfield and Mangels' (2003) proposal that the feedback related fronto-central positivity is a manifestation of an attentional orienting process, our results suggest that greater attention given to negative feedback led to better learning outcomes. This pattern of the association between the P3a and learning found in our sample resembles that found in adults (Ernst and Steinhauser, 2012; Arbel and Wu, 2016). More specifically, in Arbel and Wu (2016), large P3a to negative feedback were associated with strong learning (Arbel and Wu, 2016). Interestingly, smaller P3a to positive feedback was found related to smaller number of errors committed before reaching a learning criterion. It is possible that for fast learners in our sample positive feedback became more confirmatory than informative early in the learning process. In other words, it is likely that fast learning was associated with a more effective processing of feedback, resulting in a steeper reduction in the need to attend to positive feedback to facilitate learning. In line with these findings, Arbel and Wu (2016) reported that the amplitude of the P3a to positive feedback decreased sharply after the first block of learning, suggesting that the processing of positive feedback peaks when positive feedback first confirms the correctness of choices and becomes less important as learning progresses.

The results of the study provide evidence of a relationship between the processing of feedback in school age children and learning. The interaction between age and the FRN as predictors of learning, points to developmental changes in the processing of positive feedback even in the narrow age range of 8–11 years. The results related to the older children in the sample share similarities with patterns exhibited by adults, for whom strong learning is associated with large FRN to positive feedback and large P3a to negative feedback (Arbel and Wu, 2016). These age-related differences are in line with evidence of developmental changes in executive functions, particularly during the early school years (e.g., Welsh et al., 1991; Casey et al., 1997; Rubia et al., 2007; Vijayakumar et al., 2014).

## Data Availability Statement

The raw data supporting the conclusions of this article will be made available by the authors in accordance with the process set by the first author's institute.

## Ethics Statement

The studies involving human participants were reviewed and approved by Partners HealthCare IRB. Written informed consent to participate in this study was provided by the participants' legal guardian.

## Author Contributions

YA provided the training and oversight related to data collection and analysis, contributed to ERP data analysis, and led the writing of the manuscript. AF conducted and wrote the statistical analysis. All authors contributed to the article and approved the submitted version.

## Conflict of Interest

The authors declare that the research was conducted in the absence of any commercial or financial relationships that could be construed as a potential conflict of interest.

## Footnotes

^1^Because the FRN is a negative going component, referring to its amplitude as “larger” means that it is more negative or less positive.
